# Biosurfactant-and-Bioemulsifier Produced by a Promising *Cunninghamella* *echinulata* Isolated from Caatinga Soil in the Northeast of Brazil

**DOI:** 10.3390/ijms150915377

**Published:** 2014-09-01

**Authors:** Nadielly R. Andrade Silva, Marcos A. C. Luna, André L. C. M. A. Santiago, Luciana O. Franco, Grayce K. B. Silva, Patrícia M. de Souza, Kaoru Okada, Clarissa D. C. Albuquerque, Carlos A. Alves da Silva, Galba M. Campos-Takaki

**Affiliations:** 1 Master’s Course in Development of Environmental Processes, Catholic University of Pernambuco, 50050-900 Recife, PE, Brazil; E-Mails: nadi.elly@hotmail.com (N.R.A.S.); macluna@bol.com.br (M.A.C.L.); 2 Academic Unit of Serra Talhada-UAST, Federal Rural University of Pernambuco, 56900-000 Serra Talhada, PE, Brazil; E-Mail: andrelcabral@msn.com; 3 Department of Biology, Federal Rural University of Pernambuco, 52171-900 Recife, PE, Brazil; E-Mail: lucianafranco@terra.com.br; 4 Nucleus of Research in Environmental Sciences and Biotechnology, Catholic University of Pernambuco, 50050-590 Recife, PE, Brazil; E-Mails: grayce_kelli@yahoo.com.br (G.K.B.S.); tyttams@hotmail.com (P.M.S.); kao@unicap.br (K.O.); albqqs@yahoo.com.br (C.D.C.A.); calves@unicap.br (C.A.A.S.)

**Keywords:** *Cunninghamellaechinulata*, agroindustrial substrates, biosurfactant, bioemulsifier, spread oil, reduction in viscosity, polymeric molecule

## Abstract

A Mucoralean fungus was isolated from Caatinga soil of Pernambuco, Northeast of Brazil, and was identified as *Cunninghamella echinulata* by morphological, physiological, and biochemical tests. This strain was evaluated for biosurfactant/bioemulsifier production using soybean oil waste (SOW) and corn steep liquor (CSL) as substrates, added to basic saline solution, by measuring surface tension and emulsifier index and activity. The best results showed the surface water tension was reduced from 72 to 36 mN/m, and an emulsification index (E_24_) of 80% was obtained using engine oil and burnt engine oil, respectively. A new molecule of biosurfactant showed an anionic charge and a polymeric chemical composition consisting of lipids (40.0% *w*/*w*), carbohydrates (35.2% *w*/*w*) and protein (20.3% *w*/*w*). In addition, the biosurfactant solution (1%) demonstrated its ability for an oil displacement area (ODA) of 37.36 cm^2^, which is quite similar to that for Triton X-100 (38.46 cm^2^). The stability of the reduction in the surface water tension as well as of the emulsifier index proved to be stable over a wide range of temperatures, in pH, and in salt concentration (4%–6% *w*/*v*). The biosurfactant showed an ability to reduce and increase the viscosity of hydrophobic substrates and their molecules, suggesting that it is a suitable candidate for mediated enhanced oil recovery. At the same time, these studies indicate that renewable, relatively inexpensive and easily available resources can be used for important biotechnological processes.

## 1. Introduction

Biosurfactants were first discovered as extracellular compounds of fermentation by bacteria but which have both clearly defined hydrophilic and hydrophobic groups. Initially they were regarded as interesting due to their ability to increase the solubility of insoluble or poorly soluble hydrocarbons. They occur in nature in bacteria, yeasts, and filamentous fungi [1].

However, there are few filamentous fungi that produce biosurfactants from renewable sources. An overview of biosurfactants produced by reported bacterial species is well investigated, however relatively fewer fungi are known to produce biosurfactants [2]. Among fungi, *Candida bombicola* [3,4,5,6], *Candida lipolytica* [7,8,9], *Candida sphaerica* [10,11], *Candida ishiwadae* [12], *Candida batistae* [13], *Aspergillus ustus* [14], *Ustilago maydis* [15], and *Trichosporon ashii* [16] have been investigated. Many of these are known to produce biosurfactants from low cost raw materials. The four main classes of biosurfactant are: (a) glycolipids; (b) phospholipids; (c) lipoproteins or lipopeptides; and (d) polymeric biosurfactants. The best known glycolipids produced by these strains are sophorolipids [2].

Biosurfactants are amphiphilic surface active compounds produced by a variety of micro-organisms, with an attractive and environmentally acceptable industrial market due to their properties, and are mainly applied in enhanced oil recovery [17].

Biosurfactants are considered superior to chemically synthetic products due to their origin, structural diversity, greater substrate selectivity, low critical micelle concentration, biodegradability and low toxicity. In recent years, these advantageous features and biomolecules have gained prominence in the therapeutic and biomedical sector, and are used in pharmacological and dermatological products, and in the agricultural, food and cosmetics industries [18,19]. Biosurfactants have properties such as antimicrobial activity, and anti-adhesive activity against pathogenic microorganisms [20,21].

The chemical compositions of biosurfactants are described as glycolipids, lipopeptides, phospholipids, fatty acids, lipopolysaccharides, protein complexes, neutral lipids, and polymers [19,22].

Biosurfactants are considered extracellular secondary metabolites or associated with the cell membrane, the structure of which depends on the ratio of carbon and nitrogen sources. This has a strong influence on total production [21,23].

Patents on producing biosurfactants at an industrial level have increased, as have those that exploit the biodiversity of microorganisms for biotensioactive production, or to produce bioemulsifiers from renewable sources [20,24,25].

However, the use of these biomolecules is limited since the cost of production is high, and the production potential is low [9,26,27]. In order to raise the productivity of biosurfactant, several researchers have suggested: optimizing the bioprocess; statistical methods; using low cost, raw materials; and noted that greater availability of waste varies from country to country [25,28,29].

Thus, the literature affirms that the successful production of a tensioactive material depends on the use of renewable substrates from biotechnological processes as this cuts total costs by around 50%, despite the purification process being another obstacle to producing these compounds from a microbial origin [11,30,31,32].

In this study, an isolate of indigenous fungus from Caatinga soil of Pernambuco, Brazil is shown to be capable of producing a new biosurfactant/bioemulsifier molecule. The isolated fungus was identified by morphological and physiological studies and biosurfactant/bioemulsifier production was investigated using corn steep liquor (CSL) and soybean oil waste (SOW) which are agroindustrial wastes. In addition, some properties of the biosurfactant/bioemulsifier were investigated to confirm what the commercial prospects are and their applicability.

## 2. Results and Discussion

### 2.1. Description of the Isolate of Cunninghamella echinulata

The isolated fungus from caatinga soil was seen to have pale cream to light brown, cottony colonies; its sporophores present primary monopodial, pseudoverticilate, rarely verticillate branches; Long and short ramifications can arise from the same sporophore, which can form single branches or be branched several times. Two types of sporangioles were observed: (1) subhialines or hyaline, ellipsoid equinulates with some punctates in one end, 8.5 to 17.5−21.5 × 7 to 13.5−15.5 µm or globose (8−14.5 µm in diameter); (2) sporangioles, in common, only globose, 8.5 to 19.5−27 µm in diameter which were obviously equinulates and dark, giant, long, thin spines in older cultures. The fungus was identified as *C*. *echinulata* (Thaxt.) Thaxt. ex Blakeslee, as per Zeng and Chen [33].

**Figure 1:**
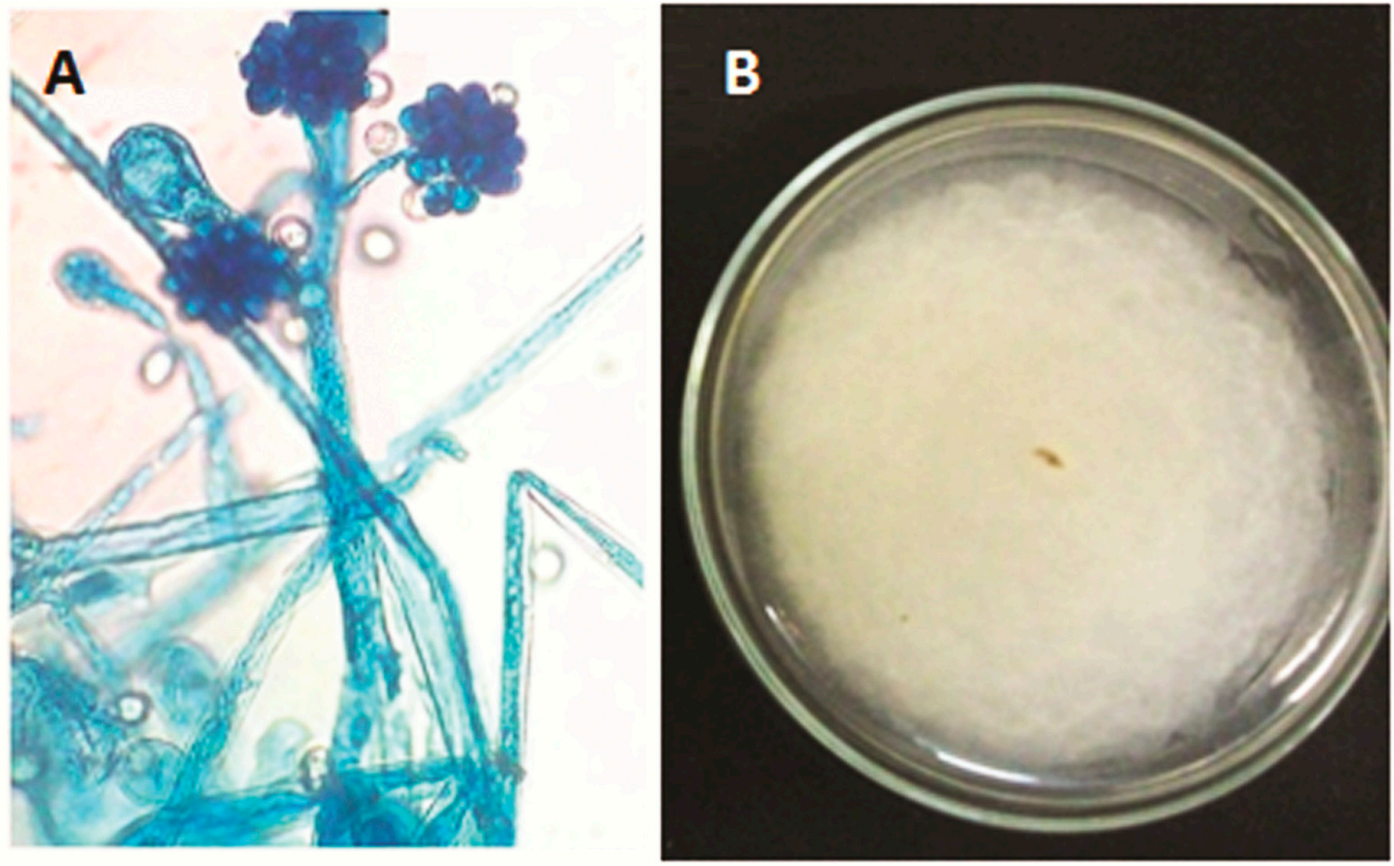
Light microscopic and macroscopic characteristics of *Cunninghamella echinulata* isolated from Caatinga soil of Northeastern Brazil. Photographes of light microscopy (**A**) and growth on Sabouraud dextrose agar (**B**).

*Cunninghamella echinulata* colonies display fast-growing at temperatures of 28 to 45 °C, develop well at pH 4, 7 and 9, and have a salinity level of up to 15%. The growth of the colony has a velvety appearance with a white coloring, as does the reverse of the colony. The light microscopy culture showed hyphae without septum, many vesicles, and single and short sporophores; its features can be observed in Figure 1A,B and these data are in agreement with that found in the literature [34,35].

### 2.2. Time-Course of Growth, Carbohydrate Consumption, pH, and Biosurfactant Production

In this study, two residues—corn steep liquor (CSL) and soybean oil waste (SOW)—were used as carbon and nitrogen sources in the saline solution to produce biosurfactant by *C. echinulata*, grown within 96 h, using a full 2^2^ factorial design, with its dependent and independent variables and their values. The results demonstrated that the fungus had the ability to reduce surface tension, and the best condition was confirmed using SOW (3% *w*/*v*) and CSL (4% *w*/*v*) and NaCl (7% *w*/*v*), described in Table 1. From this selected condition, all analyses to investigate the biosurfactant production were performed.

According to the literature, there are few fungi that can be effective in reducing surface tension in relation to bacteria such as *Pseudomonas aeruginosa* and *Bacillus subtilis*, which have the most significant results with regard to reducing such tension. However, yeasts demonstrate consistent values of tensioactives [2,36]. The best biosurfactant production achieved by *Fusarium* sp was BS-8 and this reduced the water surface tension from 72 to 32 mN/m under the condition pH 7.0, temperature 30 °C [37].

The carbon and nitrogen sources, in addition to the association with the basic solution of salts and trace elements, influenced the type and properties of biosurfactants, such as biomass accumulation. The literature describes various alternative hydrophilic and hydrophobic substrates, such as additional sources of carbon and nitrogen for synthesizing biomolecules. In this study, similar conditions were used, namely SOW as hydrophobic and CSL as hydrophilic substrates [38,39,40].

The study demonstrated a profile of growth in the first 48 h, with slight modifications until the end of cultivation (Table 1). The fungus is able to use the vegetable oil (SOW) as a carbon source that provides additional effect to biomass and the process of inducing biosurfactant production. The literature reports that biosurfactant production occurred by activating the genes responsible for their biosynthesis, and also by providing fatty acids that lengthen the lipid chain in the hydrophobic domain [41].

**Table 1:** Time-course of growth, pH, carbohydrate consumption and biosurfactant production using industrial wastes (corn steep liquor—CSL and soybean oil waste—SOW).

Time (h)	Biomass (g·L^−1^)	pH	Carbohydrate Consumption (g·L^−1^)	* Biosurfactant Surface Tension (mN/m)
24	15.0	5.0	2.30	42.9
48	30.0	5.7	1.72	41.0
72	31.0	7.1	0.17	37.0
96	32.0	7.1	0.07	36.0

* Surface tension of water: 72 mN/m and the average was 65 mN/m.

The growth in the first 48 h of culture reached a maximum value of 30.0 g·L^−1^, and slightly increased thereafter until 96 h. The surface tension decreased at 48 h of growth with values less than 40 mN/m, and continuously decreased to 36.0 mN/m. The pH is a factor that also determines the production of biomolecules, scales ranged from *Candida* species 5.0, 5.7, 6.0 and 7.8. However, *Pichia anamola* and *Aspergillus* *ustus* MFS3 showed maximum production with values in 7.0 and 5.5 pH, respectively. Thus, the production of tensioactives becomes feasible if it is possible to use low cost substrates that enable the biotechnological process [2,13,14,37,38,39].

The production of the biosurfactant is also influenced by the length of incubation, and various microorganisms are able to produce at different intervals of time as observed for *Aspergillus* *ustus* after 5 days, *Fusarium* sp. BS after 8 to 15 days, and *Candida* *bombicola* after 7 to 11 days of incubation [1,37,42].

The *C. echinulata* growth in such working conditions is related to the consumption of the sources offered by SOW and CSL residues. At the same time, the production of biosurfactant showed the surface tension reduced gradually during the 96 h of cultivation (Table 1).

**Figure 2:**
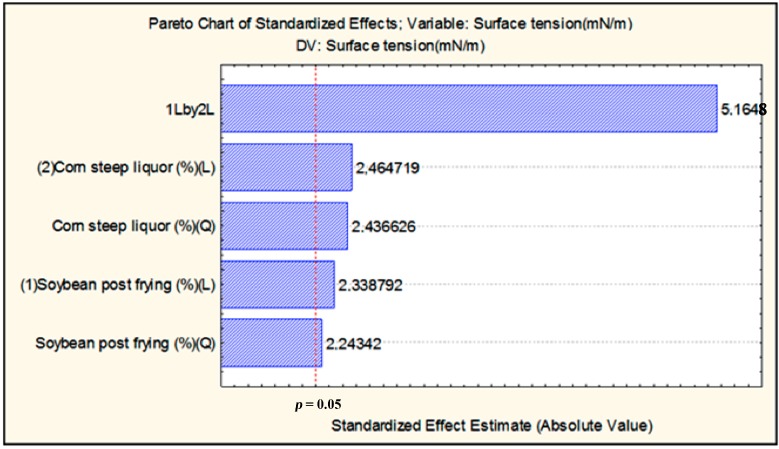
Pareto Chart of standardized effects of the cell-free broth by *Cunninghamella echinulata* after 96 h of cultivation for the 2^2^ full experimental factorial designs on surface tension. The point estimates the statistical significance (at *p* = 0.05).

Figure 2 illustrates the Pareto chart, with a 95% confidence level, for the estimated effects of CSL and SOW in reducing the surface tension. It was seen that the interaction of the two substrates CSL (hydrophilic) and SOW (hydrophobic) at 3% SOW and 4% CSL, and 7% of NaCl had a significant influence on reducing the surface tension. Both substrates were statistically significant for biosurfactant production by *C. echinulata.* These results are in accordance with the literature that affirms that physiological biosurfactant production is associated with the assimilatory mechanism in response to exposure to hydrophobic substrates [1,2,17].

### 2.3. Emulsification Characteristic of the Biosurfactant

During the past two decades, biosurfactants have been under continuous investigation as a potential replacement for synthetic surfactants. Due to their having properties such as biodegradability, environmental compatibility, low toxicity, high selectivity and specific activity at extreme temperatures, pH and salinity, they have steadily gained in significance in industrial and environmental applications such as bioremediation, soil washing, enhanced oil recovery and other general oil processing and related industries [22,23,24,25,28,30].

**Figure 3:**
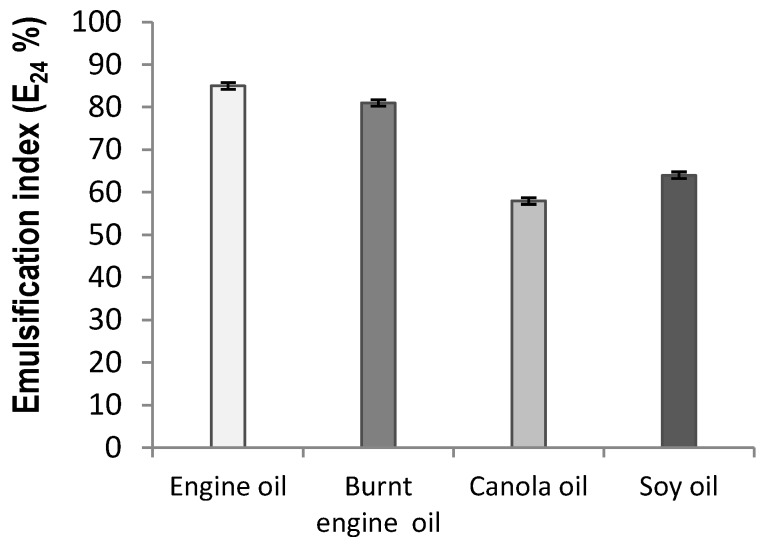
Emulsifier Index (E_24_) of the biosurfactant produced by *Cunninghamella echinulata* using corn steep liquor (CSL) and soybean oil waste (SOW) tested with engine oil, burnt engine oil, canola and soy oil.

Amiriyan *et al.* [39] suggested that emulsifier activity depends on the affinity of bioemulsifier for hydrocarbon substrates which involves a direct interaction with itself rather than an effect on surface tension of the medium. In the present study, a consistent emulsion was formed with the hydrophobic substrates of burnt engine oils and engine oils, and showed values above 80%. However, the vegetable oils of canola and soybean showed values near 60% (Figure 3). The supplementary Figure S1 was used as control of emulsification activity by culture medium; and the Figure S2 showed stable emulsion to four hydrophobic substrates using the CMC (20 g/L) of the isolate biosurfactant produced by *C.* *echinulate* forming 65%, 70%, 80% and 85% index (E_24_) to soybean oil, canola oil, motor oil and burnet motor oil, respectively.

Among the various bioactive compounds, biosurfactant (BS)/bioemulsifiers (BE) are attracting major interest and attention due to their structural and functional diversity. Our results show the bioemulsifier (BE) property of the biosurfactant (BS) produced by *C. echinulata*, a compound which compound comprises the polar and nonpolar moieties of the molecule and is able to emulsify biphasic systems (water and oil). The emulsification values obtained were compatible with those of the fungal and bacterial bioemulsifiers described in the literature [2,9,10,37,43].

### 2.4. Stability Studies

**Figure 4:**
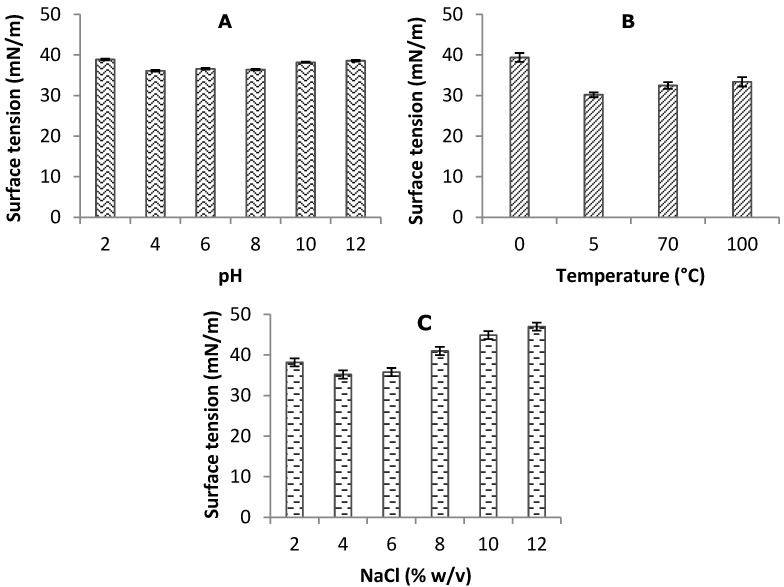
Stability of surface tension of biosurfactant produced by *Cunninghamella chinulata* using corn steep liquor (CSL) and soybean oil waste (SOW). Influence of pH (**A**); temperature (**B**); and sodium chloride concentrations (**C**) on surface tension stability.

**Figure 5:**
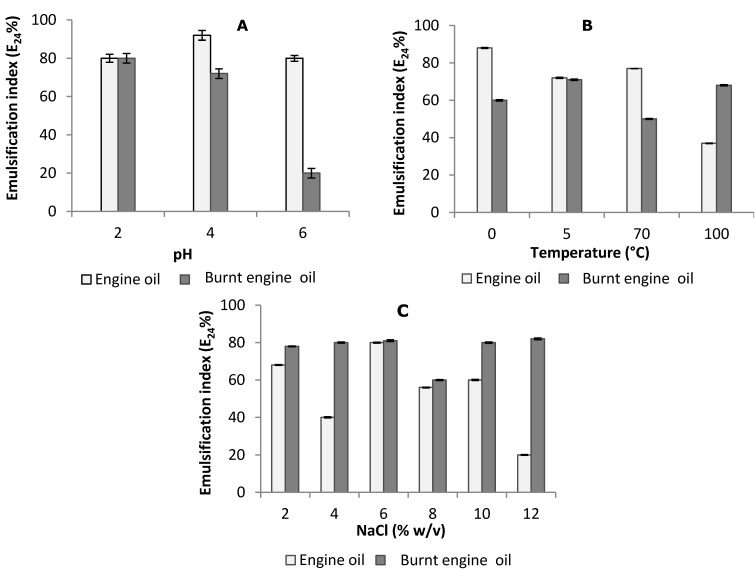
Stability of bioemulsifier of the biosurfactant produced by *Cunninghamella echinulata* using CSL (corn steep liquor) and SOW (soybean oil waste). Studies of the effect of pH (**A**); temperature (**B**); and sodium chloride concentrations (**C)** on emulsifier index (E_24_).

As observed in Figure 4A–C and Figure 5A–C, the biosurfactant stability results of some of these results are quite impressive compared to those of the most potent biosurfactant described in the literature [44,45,46].

The stability of the surface tension and emulsifier index are important factors for using biosurfactants under specific environmental conditions. The ability to reduce the surface and interfacial tension is a key parameter for detecting the production of surface-active compounds. On the other hand, the formation of emulsion is also an indicator of the emulsifier property of isolated biosurfactant [47,48,49].

The effects of temperature, pH and salinity concentration are factors that influenced the activity and stability of the emulsifier index and surface tension due to the presence of their functional groups and are important properties of the biosurfactant produced by *C. echinulata* (Figure 4 and Figure 5)*.* These results obtained for biosurfactant produced by *C. echinulata* showed that the cell-free broth was thermally stable. When the pH was gradually increased, there was a noticeable stability in the surface tension value until pH 12 was observed. The cell-free broth of *C. echinulata* was adjusted for various pH in the range 2–12 at room temperature, following which the surface activities were measured. The surface tensions remained practically uniform at all pHs, indicating that variation in pH had no appreciable effect on surface tension or the emulsifier index.

However, a small change was observed at pH 12 which must be the consequence of the desnaturation of proteinaceous compounds of the biosurfactant under extreme pH, as suggested by Ghurye *et al.* [50]. However, the variations in salt concentration values were stable at 8% and 10%. Similar behaviors regarding stability were also observed for the biosurfactants produced by *Fusarium* sp [37], *Candida lipolytica* [8], and *Candida sphaerica* [11]. Changes were observed with the increase of sodium chloride effects.

The stability of the biosurfactant *C. echinulata* at different temperatures was tested and demonstrated to be stable during one hour of incubation at 0–100 °C as shown in Figure 4B. The stability was maintained in both oils at 5 °C, using the engine oil, and showed a maximum efficiency of 80% while increasing the temperature decreases the index. However, the burnt engine oil results showed a maximum efficiency of over 60% when the temperature reached 100 °C.

At different NaCl concentrations, the biosurfactant was stable throughout the emulsification activity. Values correspond to 50% of burnt motor oil, although the engine oil showed values lower than 40% in the concentrations of 4 and 12. The major increase of biosurfactant emulsification for the two oils (engine and burnt engine) suggested that this depends on the affinity for substrates, as shown in Figure 5C. The results obtained for the emulsification index are corroborated as is the interaction of the hydrophobic portion of the biosurfactant with hydrocarbon substrates, according to Shavandi *et al.* [51].

### 2.5. Yield, CMC, Ionic Charge and Preliminary Chemical Composition of Biosurfactant

The results obtained showed a higher yield of 4.0 g·L^−1^ of isolated biosurfactant produced by *C. echinulata*. These results show that the Mucoralean fungus *C. echinulata* is an excellent tensioactive/bioemulsifier producer, when compared with fungi mentioned as producing biosurfactant from renewable sources such as glycerol, oleic acid and other wastes [1,3,52].

The chemical characterization of the isolate molecule of the biosurfactant showed the presence of lipids (40%), carbohydrates (35.2%) and proteins (20.3%). The biosurfactant showed an anionic profile by Zeta meter with −64.9 ZPmv, 4.13 μS/cm at 26.4 °C, full scale. The zeta potential determines the function of the surface charge of the particle that serves to predict and control the stability of colloidal suspensions and emulsions, and confirmed the higher values obtained, indicating good stability by repulsion between hydrophilic particles, as per the literature [45,47,53].

**Figure 6:**
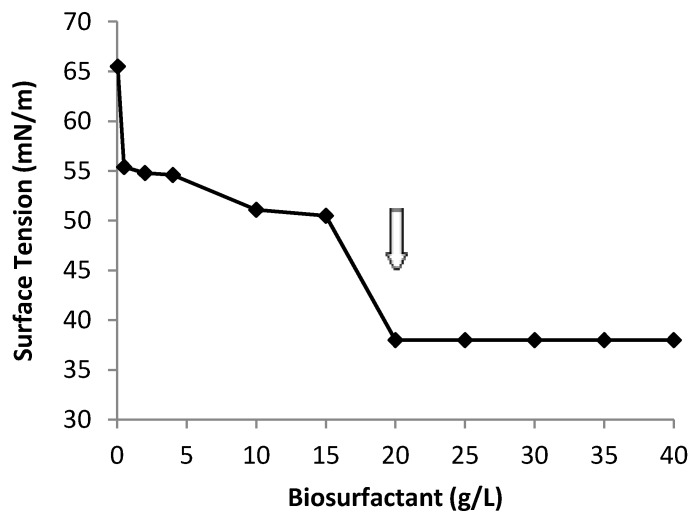
Surface tension *versus* concentration of isolated biosurfactant produced by *Cunninghamella echinulata* grown in soybean oil waste (SOW) and corn steep liquor (CSL) as substrates, added to basic saline solution on the Critical Micelar Concentration (CMC).

The critical micelle concentration (CMC) is the minimum biosurfactant concentration needed to reduce the surface tension to the maximum extent. The biosurfactant from *C. echinulata* showed a great capacity for reducing surface tension since the water surface tension was reduced from 70 to 36 mN/m when the CMC was increased to 20 g/L (Figure 6). Above this point, increasing the biosurfactant concentration did not lead to further reductions in water surface tension, thus indicating that the CMC had been reached. The results obtained show that the biosurfactant produced by *C. echinulata* possesses an increased capacity to reduce tension as compared to the biosurfactants produced by yeasts [2,8,9,10,11].

### 2.6. Oil Displacement Area-ODA

**Figure 7:**
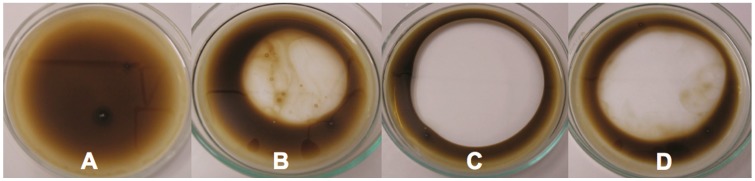
Oil displacement area formed according to substrates: (**A**) Burnt engine oil + distilled water; (**B**) Burnt engine oil + commercial detergent; (**C**) Burnt engine oil + Triton X-100 (1%); and (**D**) Burnt engine oil + isolated biosurfactant (1%) produced by *Cunninghamella* *echinulata*.

Figure 7A–D shows oil displacement area results. These results indicated a low area was formed when water was added, corresponding to 0 (Figure 5A), using commercial detergent resulted in 19.52 cm^2^ (Figure 5B), the use of Triton X-100 (1%) in 38.46 cm^2^, (Figure 5C), and the application of the biosurfactant produced by *C. echinulata* (1%) in a 37.36 cm^2^ oil displacement area (Figure 5D). However, there is little information about oil displacement areas (ODA) brought about by biosurfactants produced by filamentous fungi in the literature. However, *Bacillus* spp. (SH 20 and SH 26) were grown in ISM medium supplemented with vegetable oils but this failed to produce active biosurfactants, while in the presence of molasses they were able to produce surfactants of 51.2 ± 3.5 and 32.1 ± 2.7 cm^2^ ODA, respectively. The same authors reported that Pseudomonas aeruginosa, grown in ISM medium supplemented with waste oil, was able to produce biosurfactant and an ODA corresponding to 45.3 ± 2.8 cm^2^ [54].

### 2.7. Effect of the Biosurfactant on Viscosity

We determined the viscosity in Engine oil, Burnt Engine oil, Diesel, Biodiesel, Canola oil, Corn oil, Soybean oil, Soybean waste oil, Sunflower oil, Palm oil, Rice oil, Mineral oil and Water (Control) isolate and after adding biosurfactant isolate from *C. echinulata*. The centipoise (cP) and percentage (%) of the hydrophobic substrates was determined before and post solubilisation by using biosurfactant at 1% solution for 30 min. The best results were achieved when biosurfactant was used. Viscosity decreased as follows: Engine oil from 736.6 to 179.0 cP; Diesel from 159.1 to 43.8 cP; Canola oil from 374.0 to 110.9 cP, and Castor oil from 187.9 to 185.4 cP (Table 2).

**Table 2:** Influence of the biosurfactant produced by *Cunninghamella echinulata* in the viscosity of hydrophobic substrates

Hydrophobic Substrates	Viscosity without Biosurfactant		Viscosity with Biosurfactant	
	(cP)	(%)	(cP)	(%)
Engine oil	736.6	59.9	179.0	72.5
Burnt engine oil	148.9	48.3	210.7	87.9
Diesel	154.1	25.1	43.8	35.7
Biodiesel	36.0	29.3	51.3	41.9
Canola oil	374.0	60.8	110.9	78.5
Corn oil	47.6	38.8	404.0	66.1
Soybean oil	472.8	38.2	970.1	38.9
Soybean waste oil	380.1	61.9	556.3	45.3
Castor oil	187.9	75.1	185.4	61.6
Sunflower oil	355.5	57.6	493.3	40.9
Palm oil	403.0	67.7	536.3	45.1
Rice oil	459.9	74.8	461.1	75.2
Mineral oil	27.0	22.0	102.2	83.3
Water(Control)	0.9	0.9	1.3	1.1

The viscosity increased after adding biosurfactant as follows: Burnt engine oil from 148.9 to 210.7 cP; Biodiesel from 36.0 to 51.3 cP; Canola oil 374.0 to 110.9 cP; Corn oil from 47.6 to 404.0 cP; Soybean from 472.8 to 970.1 cP; Soybean waste oil from 380.1 to 556.3 cP; Sunflower oil from 355.5 to 493.3 cP; Palm oil from 403.0 to 536.3 cP; Rice oil from 459.9 to 461.1 cP; Mineral oil from 27.0 to 102.2 cP and Water as Control. And sometimes the increase observed is justified because of the oils’ properties, since they are consistent donors. Accordingly, the result of the biosurfactant produced by *C. echinulata* for cleaning oil contaminated plates is described. The results indicate two mechanisms that increase and decrease the viscosity using hydrophobic substrates when the new biosurfactant solution is used at CMC (20 g/L). The viscosity results suggest the new biosurfactant is a candidate for mediated enhanced oil recovery and other applications when it is necessary to increase viscosity and this is in accordance with the literature [55,56].

## 3. Experimental Section

### 3.1. Isolation, Identification and Preservation of the Microorganism

The isolation of the microorganism from Caatinga soil of the Pernambuco, Northeast of Brazil was carried out during the summer period. The isolation was performed using the successive dilution method soil/water (10^−1^, 10^−2^, 10^−3^ and 10^−4^). The isolation of fungi was carried out using media: Marttin (1.0 g K_2_HPO_4_, 0.5 g MgS0_4_; H_2_O, 5.0 g peptone, 10 g dextrose; 0.03 g Rose Bengal; 16 g agar to 1000 mL of distilled water, with chloramphenicol 0.008%); Sabouraud dextrose agar (peptone 5 g, glucose 20 g, 15 g agar, 1000 mL distilled water, pH 6.5). Plates were incubated at 28 °C until colonies appeared and were then counted. The purified colonies were transferred to new Petri dishes containing a similar medium and incubated at 28 °C, at 5 days. After this period, the monosporic culture was transferred to test tubes containing Sabouraud dextrose agar and preserved at 5 °C. Each experiment was carried out in triplicate. Identification was made using the key to Mucoralean fungi given by [37]. The isolated and identified fungus was preserved by freeze-drying and freezing in glycerol solution.

### 3.2. Agroindustrial Substrates

The agroindustrial residues were kindly provided from Corn Products, (Cabo, PE, Brazil) (corn steep liquor-CSL) and from street stalls that sell hot food in Recife-PE (soybean waste oil-SOW).

### 3.3. Culture Conditions and Biosurfactant Production

The isolate of the fungus was inoculated in Petri dishes containing Potato dextrose agar medium and incubated at 28 °C for 72 h until sporulation. After this period, the sporangioles were counted (10^7^/mL) using a hematimeter chamber, and aliquots of 5% (*v*/*v*) suspension were inoculated into 250 mL Erlenmeyer flasks containing 100 mL of the medium for biosurfactant production (KH_2_PO_4_ 2.0 g, MgSO_4_ 1.0 g) and 1 mL/L elements of trace solution was added (FeSO_4_·7H_2_O 0.63 mg, MnSO_4_ 0.01 mg, ZnSO_4_ 0.62 mg/L, pH 5.0). The substrates of soybean waste oil (SOW) and corn steep liquor (CSL) were used as agroindustrial substrates, according to factorial designs 2^2^. The flasks were incubated in an orbital shaker at 150 rpm, at 28 °C at 96 h. The cell-free metabolic liquid was obtained from centrifugation at 10,000× *g* for 15 min, and then filtered.

### 3.4. Kinetics of Growth and Biosurfactant Production

The growth of strains of *C. echinulata* was accompanied by a biomass and results were expressed in (g/L). Aliquots were collected at defined intervals of time and submitted to analysis to determine the biomass, total carbohydrate consumption, pH, surface tension, the stability of bioproducts, and to extract and characterize the biosurfactant. All experiments were performed in triplicate.

### 3.5. Determining the pH

Aliquots of the cell-free metabolic liquid were used to determine pH using an Orion potentiometer (Model 310) (Orion Research Inc., Cambridge, MA, USA).

### 3.6. Determining the Surface Tension

The surface tension was monitored for biosurfactant production from the cell-free metabolic liquid using a Tensiometer model Sigma 70 (KSV Instruments Ltd., Helsinki, Finland) by the Du Nouy ring method at room temperature (±28 °C), as per Kuyukina *et al.* [57]. Measurements of surface tension from distilled water and from the conventional medium were used as control.

### 3.7. Determining the Emulsification Index and Activity

To determine the percentage of emulsification, the samples removed after 96 h fermentation were centrifuged at 10,000× *g* for 15 min and then analyzed according to the methodology described by Cooper and Goldenberg [58]. 1.0 mL of hydrophobic substrates and 2 mL of cell-free metabolic liquid containing the biosurfactant was added to a graduated tube, and the mixture was vortexed for 2 min. Hydrophobic substrates of engine oil and burnt engine oil were tested. The percentage of emulsification (E_24_) index is given as percentage of height of emulsified layer (mm) divided by total height of the liquid column (mm).

### 3.8. Determining the Critical Micelle Concentration (CMC)

The critical micelle concentration of biosurfactant isolated during 96 h of cultivation was determined in aqueous solution. The surface tension was measured on an automatic tensiometer (model Sigma 70 KSV Ltd., Helsinki, Finland) using a platinum-iridium ring. The sample of the isolated biosurfactant was diluted in different concentrations, starting with a minimum concentration of 0.001 mg/mL until reaching the CMC was seen to have produced a surface tension of a constant value. A graph of the percentage concentration of biosurfactant (%)/TS (surface tension) was plotted.

### 3.9. Determining the Stability of the Biosurfactant

Stability studies were undertaken using the cell-free broth obtained after centrifuging the cultures at 10,000× *g* for 15 min. Four milliliters of this broth were heated at 0, 5, 70, and 100 °C for 1 h, and cooled to room temperature, after which the surface tension and emulsification activity were measured. The surface tension and emulsification index of the broth were also determined after exposure at the same temperatures. To study the pH stability of the cell-free broth, its pH was adjusted to different pH values (2–12) and the surface tension and emulsification activity were measured. The liquid-culture pH was adjusted with 1 M NaOH. The effect of NaCl concentrations (2%–12%) on reducing the surface tension and emulsification capacity of the cell-free culture broth were also determined. The tests were performed in triplicate.

### 3.10. Isolation of the Biosurfactant

The biosurfactant produced by *C. echinulata* was isolated by the precipitation method using the cell-free metabolic liquid with acetone 1:1 (*v*/*v*) as per Paraszkiewicz *et al.* [20]. The precipitate was allowed to stand for 24 h at 4 °C, and after this period was centrifuged at 4000 rpm for 15 min, at 5 °C. The supernatant was discarded and the isolated biosurfactant was submitted to dialysis against deionized water, which was changed every 3 h, for 96 h at 5 °C. The biosurfactant was collected and freeze-dried [51].

### 3.11. Characterization of the Biosurfactant

Protein content in the isolated biosurfactant was estimated by total protein test kit from Labtest Diagnostica S.A., Minas Gerais, Brazil. The total carbohydrate content was estimated by the phenol-sulphuric acid method [59]. The lipid content was determined according to Manocha *et al.* [60].

### 3.12. Ionic Charge and Viscosity

The ionic charge of the biosurfactant was determined by using a Zeta potentiometer model ZM3-D-G, Zeta Meter System 3.0+, with direct images to the video of the Zeta Meter, San Francisco, CA, USA. The top row was filled with a pure compound of a known ionic charge. The substance is known as anionic sodium dodecyl sulfate (SDS) at a concentration of (0.02 M) cationic substance and barium chloride (0.05 M). The Petri dish was kept at room temperature for 48 h. The result was calculated when the precipitation lines appear, as per Meylheuc *et al.* [61].

### 3.13. Oil Spread Test (Oil Displacement Area-ODA)

The oil spreading test was carried out using the methodology described by Diab and El Din [54] in order to evaluate the effect of the biosurfactant on the oil displacement area. Twenty milliliter of burnt engine oil was placed on each Petri dish (9 mm diameter), and this was followed by spreading 1 mL of distilled water (A), 1 mL of commercial detergent (B), 1 mL of synthetic surfactant Triton X-100 (C), and 1 mL of crude biosurfactant produced by by *Cunninghamella* *echinulata* (D) in the centre of a film of oil. The Petri dishes were incubated at room temperature (28 °C) for 24 h. After this period, the diameters of clear zones of the assays were measured in cm^2^. Distilled water was used as control. All data were reported as the range of triplicate samples. The diameter of the clear zone formed was calculated as an oil displacement area (ODA) according to Morikawa *et al.* [62] using the following equation [60]: ODA = 22/7 (radius)^2^ cm^2^.

### 3.14. Determining Oil Viscosity

In order to investigate the effects that the biosurfactant produced on the viscosity, tests were carried out at a fixed volume of 6 mL. These viscosities were measured at 25 °C using a standard viscometer (Brookfield (Middleboro, MA, USA) TC 500). The hydrophobic substrates of Engine oil, burned engine oil, Diesel, Biodiesel, Corn oil, Coconut oil, Mineral oil, Soybean oil, Soybean post-frying oil and water as control were tested. In the same sequence as the hydrophobic substrates we added 2 mL of biosurfactant solution at 1.0% (*w*/*v*), which was vortexed for 1 min. and the viscosity was measured again. The viscosity results were expressed in cP and %.

### 3.15. Statistical Analysis

In order to verify the effect of independent variables (the concentrations of industrial residues, corn steep liquor-CSL and soybean oil waste-SOW) on biosurfactant production, a complete factorial design 2^2^, with three central values, was used (Table 3). The results were then examined to determine the main effects of all factors and tests were performed to determine which factors are statistically significant. Analysis and graphs of the data, and all calculations were performed using the Statistica version 7.0 software package (Statsoft Inc., Tulsa, OK, USA), and the significance of the results was tested at (*p* ≤ 0.05).

**Table 3:** Level and factors applied to the factorial design compound 2^2^.

Parameters	(−1.68)	(−1)	0	(+1)	(+1.68)
Soybean oil waste (SOW) (%)	0.62	3.00	6.50	10.00	12.38
Corn steep liquor(CSL) (%)	2.64	4.00	6.00	8.00	9.36

## 4. Conclusions

The new biosurfactant produced by the Mucoralean fungus *C. echinulata*, besides being a good surfactant, has attractive properties as a tensioactive and emulsifier compound. The biosurfactant has several properties and it is not affected by temperature, pH, nor the sodium chloride concentration. The preliminary biochemical composition suggests that the molecule is a new polymeric biosurfactant, comprising lipids (40%), carbohydrates (35.2%) and proteins (20.3%). The biosurfactant induced a larger oil displacement area using burnt engine oil, and reduced viscosity. These are characteristics that are desirable for industrial processes, given that it has several properties which could be attractive and is a potent emulsifier and surface-active compound which are useful in many fields of industry. This approach to the biosurfactant produced by *C. echinulata* represents a promising, simple and cost effective tool for treating the oily sludge generated during the periodical cleaning of oil storage tanks and by other petroleum industries, and has other applications when an increase in viscosity is necessary.

## Supplementary Materials

Supplementary materials can be found at http://www.mdpi.com/1422-0067/15/9/15377/s1.

## Supplementary Files

Supplementary File 1
