# Supplementary material for: Biosurfactant-and-Bioemulsifier Produced by a Promising Cunninghamella echinulata Isolated from Caatinga Soil in the Northeast of Brazil
Source: Int J Mol Sci. 2014 Sep 1;15(9):15377–95. doi: 10.3390/ijms150915377 (PMC4200836; doi:10.3390/ijms150915377)

Supplementary Information

The medium for biosurfactant production in the best condition was used the interaction of 4% of corn steep liquor (CSL), soybean oil waste (SOW), and 7% of sodium chloride had a significant influence on reducing the surface tension, as well as emulsification activity,

Figure S1. Absence of emulsification activity index (E_{24}) of the medium used for biosurfactant production in the best condition of the factorial design. A, Burnet motor oil; B, Motor oil; C, Canola oil; D, Soybean oil.

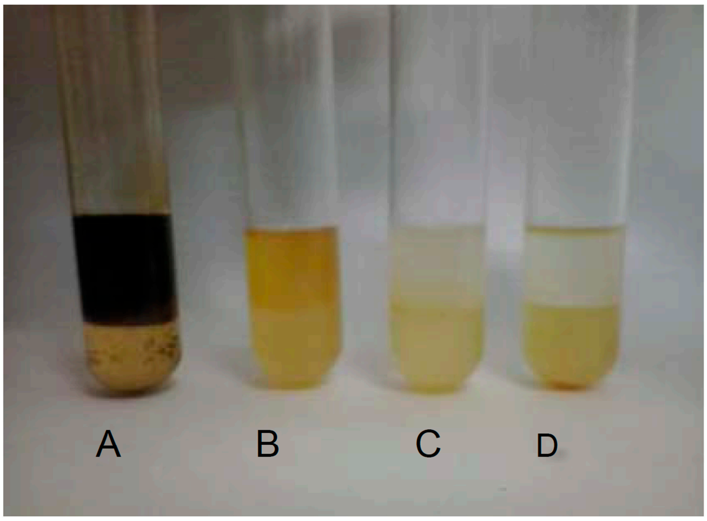

The cell-free broth obtained after centrifuging of the cultures of *Cunninghamella echinulate* was tested the emulsifying activity using different hydrophobic substrates. The results showed higher and stable emulsifying activities from four different hydrophobic substrates using the CMC (20 g/L) of the isolated biosurfactant produced by *C. echinulate* corresponding from 65%, 70%, 80% and 85% to soybean oil, canola oil, motor oil and burnet motor oil, respectively.

Figure S2. Emulsification activity index (E_{24}) using the CMC concentration (20g/L) of the biosurfactant produced by *Cunninghamella echinulate*. A, Burnet motor oil; B, Motor oil; C, Canola oil; D, Soybean oil.

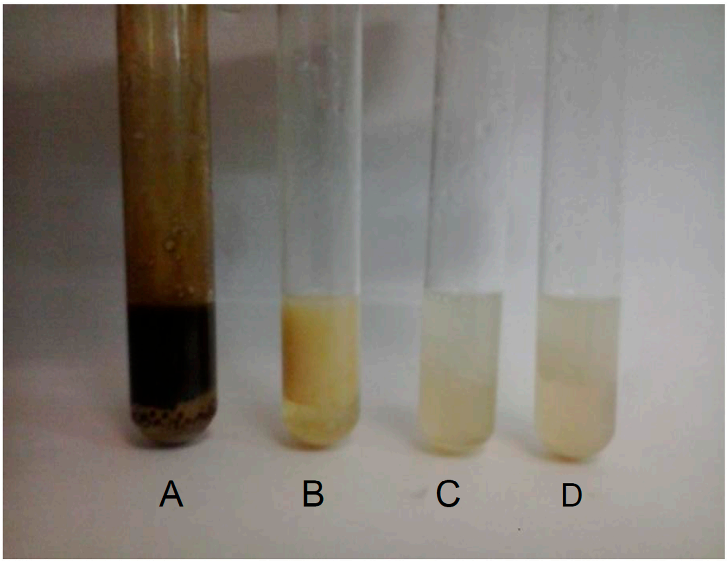

Supplement: Supplementary File 1 [file ijms-15-15377-s001.pdf]
